# Quantifying the emergence of moral foundational lexicon in child language development

**DOI:** 10.1093/pnasnexus/pgae278

**Published:** 2024-08-20

**Authors:** Aida Ramezani, Emmy Liu, Spike W S Lee, Yang Xu

**Affiliations:** Department of Computer Science, University of Toronto, Toronto, ON M5S 3G4, Canada; Language Technologies Institute, Carnegie Mellon University, Pittsburgh, PA 15213, USA; Rotman School of Management, University of Toronto, Toronto, ON M5S 3E6, Canada; Department of Psychology, University of Toronto, Toronto, ON M5S 3G3, Canada; Department of Computer Science, University of Toronto, Toronto, ON M5S 3G4, Canada; Cognitive Science Program, University of Toronto, Toronto, ON M5S 3H7, Canada

**Keywords:** morality, language, child development, moral emergence, computational analysis

## Abstract

Theorists have argued that morality builds on several core modular foundations. When do different moral foundations emerge in life? Prior work has explored the conceptual development of different aspects of morality in childhood. Here, we offer an alternative approach to investigate the developmental emergence of moral foundations through the lexicon, namely the words used to talk about moral foundations. We develop a large-scale longitudinal analysis of the linguistic mentions of five moral foundations (in both virtuous and vicious forms) in naturalistic speech between English-speaking children with ages ranging from 1 to 6 and their caretakers. Using computational methods, we collect a dataset of 1,371 human-annotated moral utterances and automatically annotate around one million utterances in child-caretaker conversations. We discover that in childhood, words for expressing the individualizing moral foundations (i.e. Care/Harm, Fairness/Cheating) tend to emerge earlier and more frequently than words for expressing the binding moral foundations (i.e. Authority/Subversion, Loyalty/Betrayal, Purity/Degradation), and words for Care/Harm are expressed substantially more often than the other foundations. We find significant differences between children and caretakers in how often they talk about Fairness, Cheating, and Degradation. Furthermore, we show that the information embedded in childhood speech allows computational models to predict moral judgment of novel scenarios beyond the scope of child-caretaker conversations. Our work provides a large-scale documentation of the moral foundational lexicon in early linguistic communication in English and forges a new link between moral language development and computational studies of morality.

**Significance Statement**

Research in moral psychology has suggested that morality is built on foundational modules. When do these foundations emerge in life? We conduct a large-scale computational analysis to identify the timeline in which English-speaking children begin expressing moral foundations through speech with their caretakers. Our analysis reveals that children start communicating moral concerns about care and fairness earlier than authority, loyalty, and purity. We observe variations in how often children and their caretakers express the positive and negative aspects of moral foundations. We also show how moral linguistic information in childhood speech helps machines predict moral judgment of novel scenarios. Our work sheds light on the emergence of the English moral lexicon and connects moral language development with computational moral inference.

## Introduction

An influential modern theory of morality, Moral Foundations Theory, suggests that moral judgments are intuitive and driven by modular foundations (1). Each foundation involves two polarities representing the positive and negative aspects of morality: Care/Harm, Fairness/Cheating, Authority/Subversion, Loyalty/Betrayal, and Purity/Degradation.^a^ Among the extensive literature that describes and discusses moral foundations (3–16), one important issue remains, namely when do people begin to communicate moral foundations in life? Here we offer, to our knowledge, the first computational investigation into this question through the lens of natural language in childhood speech.

Recent research has shown that language or language use preserved in text corpora reflects people’s moral concerns and sentiments, though imperfectly. For instance, computational models trained on textual corpora can inform about the moral foundations that people refer to when discussing their political and social concerns (17–22). However, such text-based approaches to morality have not previously been used to probe the emergence of moral foundations in child development. Our goal here is to connect this line of work with a separate strand of research on moral developmental psychology toward understanding the emergence of moral foundations.

**Fig. 1.**
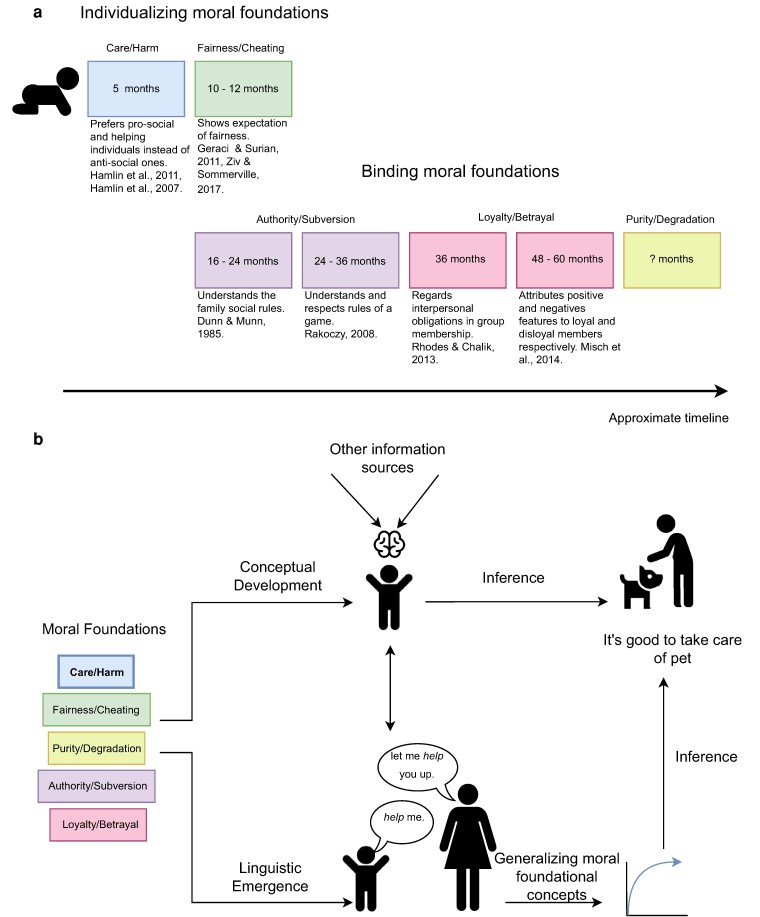
A summary of the related work and our framework. a) The order of conceptual development of moral foundations in children as inferred from the moral development literature. b) Models based on childhood speech concerning moral foundations are used to automatically infer people’s moral judgment toward novel scenarios.

Research in developmental psychology has explored the development of specific moral senses in children (23–29). In particular, researchers have examined when children become sensitive to or develop an understanding of morality within both controlled experimental environments and naturalistic contexts. Drawing upon Moral Foundations Theory as the basis of our investigation, here we summarize the previous work and outline a rough timeline for the emergence of moral foundations based on the existing literature. This summary represents the earliest observations of moral behavior in children in previous studies. However, it is noteworthy that children’s ability to evaluate the moral rightness or wrongness of different acts may develop later (30). We visualize the timeline in Fig. 1a and describe the details of the findings about the five moral foundations as follows.

### Care

The preference for prosocial behaviors (e.g. helping an activity) over antisocial ones (e.g. hindering an activity) is an example that reflects the emergence of the Care moral foundation. It has been shown that children as young as 5–6 months old prefer helping agents over hindering ones (31–33).

### Fairness

Children tend to respect and prioritize equal distribution of resources (34–36). This sensitivity to fairness has been observed in children with an average age of 15 months, who show an expectation of a fair distribution rather than an unfair one (37), and prefer fair agents over unfair agents (38–40). By repeating similar experiments with infants in different age groups (6 months, 9 months, 12 months, and 15 months), studies have suggested that 9 months of age is the transitional period when infants start to develop expectations for fairness, and the first signs of this moral foundation are observed after this stage (41).

### Authority

Observations of young children’s behavior and language during family conflicts suggest that children as young as 16 months can anticipate that certain actions are prohibited by their mothers, which suggests an emerging understanding of authority and family rules in the second year (42). Gaining awareness of the caregiver’s authority itself can be even expected to develop earlier than this time, as a result of the children’s exposure to family conflicts (43).

Related studies further show that children aged 2 to 3 years show receptiveness to authority, as they respect the rules when playing games and protest against rule breakers (44). Studies involving children aged four and older have also reported their capacity to perceive and respond to authority, with responses varying based on the age and position of the person giving commands in various social contexts (45–47).

### Loyalty

Loyalty is observed to emerge after the third year of life in childhood. The development of loyalty in children is reflected in several findings, such as the ability of 4-year-olds and above to keep secrets of the group (48), the tendency to attribute positive and negative adjectives to loyal and disloyal members respectively (49), and the expectation of out-group hostility (50). Moreover, it is found that 3-year-olds view harm within-group as violating intrinsic group obligations (51).

### Purity

The Purity foundation is mainly studied as a form of moral disgust in child development. Studies in this area suggest that moral disgust reactions, compared to other moral foundations, emerge later in development (27). For example, it has been found that children aged 6, 8, and 10 years old consider moral violations as “disgusting” more often than other negative activities (52). A recent study on children 4, 6, and 8 years old also found that the feeling of disgust toward moral violations could only be observed among the 8-year-olds (53).

What makes studying purity in child development challenging is that purity is not solely concerned with how people treat one another, but it is also associated with the moralization of body and bodily activities, hence the label “the odd corner of morality” (54). Evolutionary views on morality suggest that purity concerns, augmented with the feeling of disgust, may have evolved as a mechanism to prevent early humans from touching polluted substances and stop the transmission of diseases (54, 55). However, defining purity based on a single set of bodily activities and disgust reactions might mask its heterogeneous nature (56), which not only includes physical contamination but also any sort of spiritual contamination, such as corruption or imperfection, that might deviate humans from living a pure and noble life (5, 57). With these considerations, our summary in Fig. 1a leaves the emerging period of Purity/Degradation blank because even though children begin to experience disgust after age 3 (58), experiencing moral disgust has thus far only been observed in older children. Moreover, questions about the emergence of understanding the virtuous side of purity (not degradation) in children remain unanswered.

By gathering the empirical evidence from the moral development literature, we can construct an approximate timeline for the conceptual emergence of moral foundations, as shown in Fig. 1a. Care/Harm and Fairness/Cheating, which are considered individualizing foundations due to their focus on autonomy and benefits to individuals, are typically observed earlier than Authority/Subversion, Loyalty/Betrayal, and Purity/Degradation, which are known as the binding foundations due to their emphasis on benefits to communities and groups (17).

This estimated timeline, however, is limited in two main respects. First, despite the importance of incorporating naturalistic methods in developmental studies (59), the existing work tends to more often rely on experimental settings and restrict to only one particular moral foundation without offering a comprehensive account of all moral foundations under similar settings. For example, the available empirical evidence has documented looking and reaching preferences with infants for Care/Harm foundation (31–33) and more explicit judgments with older kids for Authority/Subversion foundation (42, 44–47). However, looking and reaching preferences and explicit judgments of relevance are very different measures and require different levels of cognitive sophistication. Moreover, since child-caregiver transgressions could take place in the first year (43), children’s awareness of their parent’s authority may begin to develop earlier as well. Therefore, the proposed timeline constructed from the past literature is approximate rather than exact and can only serve to guide further investigation of child moral development.

Second, each moral foundation has both positive and negative aspects, and whether they manifest themselves differently through the developmental time course is not clear. Studies have explored punishing unfairness and rewarding fairness (40), helping vs. hindering (31, 33), and group loyalty vs. betrayal (49, 60, 61). These approaches differ in experimental aspects, making their findings hard to compare for a coherent understanding of the developmental emergence of different moral foundations.

Previous studies have proposed two main accounts on the origins of morality. The nativist viewpoint proposes that children possess an innate understanding of moral concepts, demonstrated by infants’ responsiveness to and preferences of moral concepts like helping or fairness (27, 62, 63). Conversely, the constructivist viewpoint argues that the preferences seen in young children stem from their reciprocal interactions with the environment, rather than revealing an innate capacity to make moral evaluations. In this view, children gradually comprehend morality through active involvement in interpreting, abstracting, and assessing their social experiences (28, 30, 64). With regard to the origins of moral foundations, the Moral Foundations Theory proposes that the “first draft” of moral foundations is innate and develops over time via children’s engagement with the social and cultural dynamics of their environment (1, 5, 54). Here, instead of focusing on the origins of morality, we investigate the role of child-caretaker conversation—an important form of communication and nurturance in early life—in the growth of moral language in childhood pertaining to the moral foundations.

We develop a text-based computational approach that quantifies the emergence of moral foundations beyond characterizing particular foundations in isolation. We use a combination of computational tools and an experimental survey to quantify the emerging order of moral foundational lexicon, or keywords that signify moral foundations, in the first 6 years of life in English-speaking children. This age range aligns closely with the period of conceptual development of morality in childhood documented in the literature.

We also use our framework to explore how moral information embedded in childhood language can be used to grow computational models that are devoid of pre-existing moral knowledge for making moral inferences about novel scenarios. Such a model learns patterns from childhood moral language to make automatic predictions about moral scenarios that young children may not have encountered. We refer to this predictive task as (moral) generalization.

Figure 1b illustrates our framework. Children develop a conceptual understanding of moral foundations from multiple information sources, and their understanding of these foundations should appear in their conversations with others, including their caretakers. For example, children and caretakers can talk about caring by using related vocabulary such as *help* in different utterances like “help me” or “let me help you up”. We assess the extent to which computational models with input from childhood speech can automatically make moral inferences about new scenarios such as “taking care of one’s pet”, through only snippets of child-caretaker language use that are morally relevant. For this task, we also construct our models without prior knowledge of the moral scenarios that they make inferences about (i.e. blank-slate models). Overall, our work reveals previously unknown patterns of moral language development and contributes computational methods and resources for exploring moral lexicon emergence.

## The emergence of moral foundational lexicon in children

To guide our analyses, we pose two research questions about the emergence of moral foundations in child language. Our first question is based on the empirical evidence of moral development reported in the psychological literature and shown in Fig. 1a. Specifically, we investigate whether the emerging order of moral foundational lexicon in child speech mirrors the order of moral conceptual development: Care → Fairness → Authority → Loyalty (with the order of Purity being under-specified in the literature, and observing Fairness and Authority around the same time). This question is rooted in the view that children’s conceptual development and linguistic development are closely related (65, 66). It is conceivable that moral conceptual development might precede moral language development, and our current investigation does not presume that the timelines of moral conceptual development and linguistic development have to be aligned exactly. For example, children might develop early expectations for giving care and being fair in the first and second years of life, respectively (as recorded in previous studies), and thus we simply predict that they begin to use language to express the Care moral foundation before Fairness, without assuming that conceptual and linguistic development should happen around the same age. This relaxation is intended because the estimated timeline for the conceptual moral development is itself not exact.

Our second question is whether the emerging order of moral language in children follows the order in which caretakers communicate moral foundations in the linguistic environment, which might or might not match the suggested order in conceptual development. For instance, parents might choose to emphasize Purity in the linguistic input more than Fairness, which could influence the earlier emergence of the Purity moral foundation compared to Fairness in child speech. Caretakers might also emphasize negative aspects of morality (don’ts) more than positive aspects (do’s) to prevent children from wrongdoing. Our investigation of this research question is motivated by existing evidence that child-directed speech exerts a substantial influence on children’s language acquisition (67, 68). It is also consistent with Kohlberg’s view on the preconventional stage of moral development whereby children’s morality is shaped largely by adults (23).

To examine the linguistic emergence of moral foundations at scale, we use the CHILDES database (69)—one of the largest public databases of childhood speech in naturalistic settings. This corpus contains text transcripts of interactions between children and their caretakers reflecting linguistic communication for children. In total, we extracted 1,011,102 unique text transcripts, from which we extracted 384,695 utterances of Child Speech (CS) and 626,407 utterances of Child-Directed (or caretaker) Speech (CDS). See Supplementary material for the full set of sub-corpora we used for analysis.

Our study investigates the emergence of moral lexicon through linguistic appearance and frequency of moral words used in moral contexts during child and caretaker speech. Extending research on the conceptual development of morality in lab settings, this analysis helps to identify meaningful moral word usages in a naturalistic setting. To characterize when the lexicon of moral foundations emerges in child development, we collect morally relevant utterances from CHILDES drawing on a large set of words from the Moral Foundations Dictionary (MFD) (17) version 2.0 (70) which we use as the base lexicon. It includes around 2,000 English moral words that signify different moral foundations.

We study the frequency of words as a proxy for tracking their emergence in English-speaking children’s moral language for several reasons. First, frequency offers a quantifiable and reproducible measure that can be evaluated continuously through time and across diverse speech environment. Second, frequency allows us to identify when each moral word appears in childhood speech. Significance levels based on word frequency help differentiate meaningful usage from noise. Third, our approach helps reveal trends in children’s moral language through different developmental stages. Aligned with the constructivist viewpoint on cognitive moral development (30, 64, 71), frequency time courses and shifts in development offer insights into moral language expansion in childhood.

Importantly, we go beyond a purely frequency-based approach by pairing it with contextual (or moral word sense) disambiguation, which identifies word usages within moral contexts (e.g. *fair* resource allocation) and removes noisy word usages that are ambiguous and have little to do with morality (e.g. science *fair*). In other words, moral words could be used in morally irrelevant contexts due to the polysemy of words, and counting them as instances of moral language would overestimate the result. To disambiguate the context of moral words, we grouped these utterances into different clusters based on their semantic similarity. For example, utterances that use the word *fair* in a moral context (e.g. “is that fair enough?”) would end up in a different cluster from the utterance that uses this word in a nonmoral context (e.g. “did you like the fair?”). To determine which utterances are morally relevant vs. not, we conducted a survey and obtained human annotations for 670 utterances from CDS and 701 utterances from CS (see Materials and methods for details of our data collection, processing, and clustering procedures).

Table 1 provides sample utterances from each moral foundation that show a high degree of annotator agreement on moral relevance. For most of the moral foundations, the relevance of each sample utterance to the corresponding moral foundation is obvious. But the Purity/Degradation moral foundation warrants additional clarification. Consistent with ongoing debates about the nebulous meaning of this foundation (56), the sentences we classify as Purity and Degradation are mainly concerned with physical cleanliness and dirtiness and display a less obvious connection to morality than other foundations. For example, parents would often tell children that something is dirty without explicitly expressing their moral concerns. We argue that if children never learned about physical cleanliness, they would have a hard time comprehending the purity foundation in more abstract scenarios (72, 73). Moreover, the fact that our survey participants (all adults) considered these sentences to be morally relevant suggests that people might naturally think of avoiding physical dirtiness as early examples of the development of the purity foundation. Physical disgust has been shown to appear earlier than moral disgust in children’s conceptual development (52, 53, 58). Based on these observations, our work examines the emergence of both physical and moral dirtiness and cleanliness in child-caretaker conversations.

**Table 1.** Sample utterances expressing each of the five moral foundations, in virtuous and vicious forms, extracted from child speech and child-directed speech.

Moral foundation	Child-directed speech (CDS)	Child speech (CS)
Care	*we must rescue him*	*help Carrie wash dish*
Harm	*you wouldn’t hurt Adam would you*	*and they always fight*
Fairness	*is that fair enough*	*next time I’m gonna make it fair*
Cheating	*they did steal the honey*	*I’m not cheating*
Authority	*go out and tell your father you’re sorry*	*you mean she gave you permission*
Subversion	*why do you choose to be disobedient*	*except the dragon can’t even kill the knight*
Loyalty	*are you being honest with me*	*you and me do this together*
Betrayal	*do you think it was one of his enemies*	*so they got to be enemy*
Purity	*you have your own body*	*the blessing*
Degradation	*why don’t you throw that in the trash*	*nobody wiped the germ off*

Figure S1 shows word cloud plots for the moral foundational lexicon in the CHILDES dataset with respect to their frequency. These plots reveal that words such as *help*, *hurt*, *fair*, *lying*, *police*, *impolite*, *player*, *together*, *enemy*, *food*, *clean*, and *dirty* are the most frequent moral words from all foundations. Supplementary material also describes the exact number of utterances we obtained for each moral foundation from CHILDES.

To evaluate our hypotheses, we use the annotated utterances from our survey to automatically identify the moral relevance of each utterance in CHILDES and the specific moral foundation signified by it. We then track the frequency with which each moral foundation is talked about by English-speaking caretakers and children (see Materials and methods for details of our sentence annotation).

**Fig. 2.**
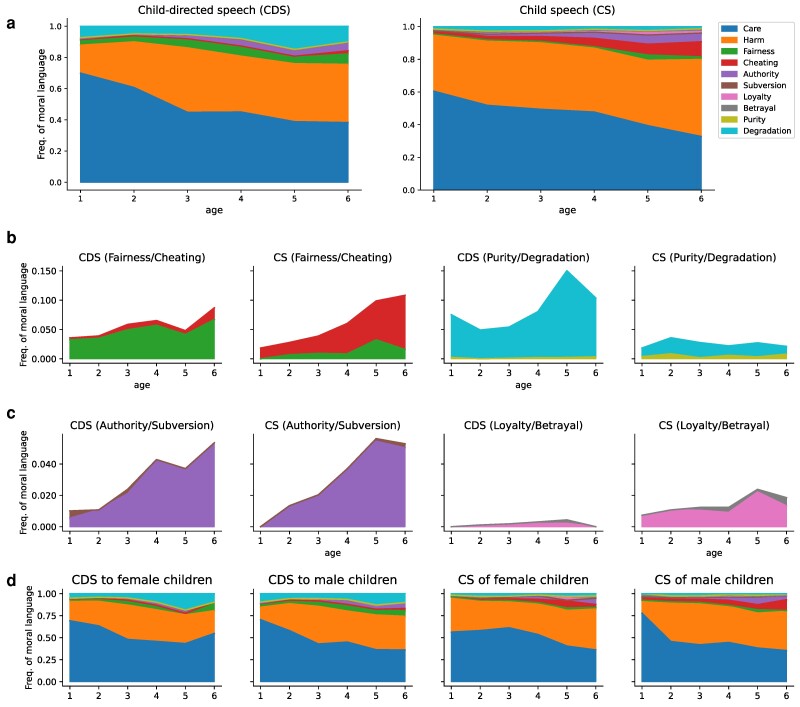
A summary of our results for identifying the emerging order of moral foundational lexicon over time. a) Stack area charts show the frequencies of moral foundational language over time. b) Frequencies of Fairness/Cheating and Purity/Degradation foundational lexicon in child-directed speech (CDS) and child speech (CS). c) Frequencies of Authority/Subversion and Loyalty/Betrayal foundational lexicon in CDS and CS. d) Stack area charts show the frequencies of moral foundational language over time within different gender groups.

Figure 2a summarizes the normalized frequencies per age group. For better visual clarity, we re-display Fairness/Cheating, Purity/Degradation, Authority/Subversion, and Loyalty/Betrayal foundations in Fig. 2b and c. A first glance at the moral foundation frequencies indicates a clear dominance of Care/Harm foundation over all the other foundations. Furthermore, both Care/Harm and Fairness/Cheating, which are known as the individualizing moral foundations, emerge earlier than the binding foundations (Authority/Subversion, Loyalty/Betrayal, Purity/Degradation) in child speech. This initial finding provides evidence for our first hypothesis, namely that the development of children’s moral language in child-caretaker conversations mirrors moral conceptual development.

Specifically, in child speech, the Care/Harm foundation is present in moral language as early as 1-year-old and contains more than 95% of moral language at that age. Fairness/Cheating foundation is the second most frequent moral foundation and gradually rises throughout child development, capturing more than 10% of moral language by age 6. Authority/Subversion is scarcely represented at age 1, but similar to Fairness/Cheating grows gradually over time and reaches 5% by age 5. We observe much less frequent usages of the Loyalty/Betrayal foundation words, not exceeding 2% of moral language by the age of 6.

A permutation test on children’s moral utterances further shows that even though some moral words appear at early ages (age 1, 2), their frequency is statistically below the average frequency of moral words, which mainly consists of words in the Care/Harm moral foundation. For example, at age 2, we identify words such as *police*, and *punish* to appear in the Authority/Subversion moral foundation and *belong* to appear in the Loyalty/Betrayal moral foundation, but compared to the rest of the moral lexicon used by 2-year-olds (e.g. *help, hug, safe*, and *hurt*), their appearance is statistically insignificant. These results suggest that children tend to use a more diverse set of moral words for the Care/Harm moral foundation, while the vocabulary used for the other moral foundations is quite sparse in early ages. Figure S2 displays the percentage of words above the significance level for each foundation and age group in child speech.

From an early age, Purity/Degradation foundation exceeds Fairness/Cheating in frequency. However, upon closer examination, most of the sentences in the Degradation foundation use the word *dirty* to describe physical dirtiness without any trace of moral disgust reactions. Utterances in the Degradation foundation that include disgust-related or spiritual impurity-related content are rare (e.g. “no you’re a spoiled brat” and “he’s so gross I can’t even tell you”). Purity utterances in CHILDES represent almost entirely children’s religious expressions, including words such as *praying* and *church*, and capture around 0.8% of moral language by the age of 6. But note that the CHILDES dataset does not differentiate between children raised in religious and nonreligious families, a distinction that we believe could have impacted the linguistic emergence of Purity words.

In summary, our results reveal the following emerging order of moral foundations in language development as manifest in speech between English-speaking children and their caretakers: Care/Harm → Fairness/Cheating → Purity (religion-related lexicon) → Authority/Subversion → Loyalty/Betrayal → (Degradation). We specifically distinguish Purity from Degradation to compare the later development of moral disgust with the earlier development of moral purity in child speech. Our finding aligns with the conceptual order of the development of morality in children, as stated in our first hypothesis, and extends the previous findings to locate the approximate emergence of Purity/Degradation foundation. All of our results are consistent across child gender when it is explicitly controlled for (Fig. 2d).

The emerging order reflected in child-directed speech (from caretakers) is similar but not identical to child speech. One notable difference is the higher frequency of the Degradation words in child-directed speech compared to child speech, which can be a result of caretakers preventing young children from disgust-related matters not perceived as disgusting by children (27, 74). We also observe that Cheating is more talked about by children, while Fairness is more predominant in caretaker speech. This asymmetry presumably reflects caretakers’ efforts to educate children about the quality of being fair. Results from ANOVA tests confirm that the rate of utterances of Degradation, Fairness, and Cheating are significantly different between child speech and child-directed speech (P<0.05).

Children also use more negation in their utterances compared to caretakers. Figures S3 and S4 show the normalized frequency of the positive and negative utterances in each moral foundation for child speech and child-directed speech, respectively. The negative utterances include at least one of the negative words *no*, *not*, and *n’t*, while the positive utterances include none. Children tend to use the negated form of the utterances in Fairness and Authority more than other foundations. For example, the negative form of the word *fair*, as in “it’s not fair” appears more frequently than its positive form in child speech. In contrast, caretakers use much less negation in their morally relevant conversations with children (except for the Purity foundation).

Our analysis in Supplementary Material and Fig. S5 suggest that the order of emergence of moral foundational lexicon is not an artifactual result of the Age of Acquisition (AoA), the age at which a word is typically learned, and the overall frequency of moral words in the CHILDES dataset. For example, even though Fairness emerges earlier than Loyalty in children’s moral lexicon, the AoA of Fairness words is not significantly different from the AoA of Loyalty words.

We also explore how children above 6 years old use the moral foundational lexicon. As demonstrated in Table S2, the number of utterances in child speech and child-directed speech for older children varies significantly by age and is notably sparser compared to the data available from younger children. Therefore, instead of analyzing the patterns for each age individually, we have combined the results from ages 7 to 11, as displayed in Fig. S11. We find that the Care/Harm moral foundation remains prominent in the moral language of older children. However, caretakers express the Fairness moral foundation more frequently than Harm, suggesting that discussions with older children may encompass new aspects of morality previously overshadowed by Care/Harm.

Measurements other than frequency can further shed light on the moral language in early development. For example, the complexity of a moral utterance would estimate the level of a child’s understanding of the moral foundation. To explore the complexity dynamics of moral language, we use the Mean Length of Utterance (MLU) as a proxy for language complexity. MLU estimates the number of words (or morphemes) in children’s sentences and is widely used as a measurement of language acquisition (75, 76). Figure S6 shows that the MLU increases over time both in CS and CDS. We also see that the moral words in Care/Harm and Purity/Degradation foundations are typically uttered in shorter sentences and Authority/Subversion contains longer sentences. The sentences in the Fairness/Cheating moral foundation are relatively longer than other foundations in younger children, but their length remains stable over time.

Up to now, our analysis has focused on the moral lexicon as the primary subject of investigation. Specifically, our findings have relied on the frequency of moral lexicon in morally relevant contexts. However, previous research has revealed that solely relying on MFD words may not uncover alternative patterns of moral language. For instance, the Care/Harm moral foundation is strongly linked with family-related, emotion-related, and health-related words, some of which are not included in the MFD (77).

To examine whether the observed patterns in the moral foundational lexicon persist in moral language excluding MFD words, we repeated our experiment on the remaining utterances in CHILDES. Since these utterances contained no MFD words, we categorized them into moral foundation and nonmoral categories by drawing on semantic similarities with our human-annotated section of the CHILDES dataset (see Materials and methods). As shown in Fig. S7, within the morally relevant non-MFD language, Care/Harm continues to dominate other moral foundations in child speech. In child-directed speech, Fairness and Authority are more prevalent compared to their frequency when exclusively considering the MFD words. Examples include a caregiver saying “sweetheart one’s enough” to implicitly refer to the Fairness moral foundation, and the sentence “Yes, you are going” to reflect parental authority over children without explicit use of the MFD words. This analysis also allows us to identify the non-MFD lexicon frequently used in moral contexts. For instance, we observe that the words *scary, mad, sick, sad, drink, monster, sticky, naughty, messy*, and *smelly* are commonly used by children in the non-MFD moral section of the CHILDES. We also identify the words *daddy, push, break, beat, hit, wrestle*, and *brother* to appear in the moral language associated with Authority in child speech. Figure S8 displays the word cloud plots for the non-MFD lexicon of the moral language in CHILDES.

Despite observing a similar pattern of results to those of the moral foundational lexicon, we exercise caution in interpreting the results of this section, mainly because our annotations were collected for sentences containing MFD words, leading to a potential mismatch in the training (MFD sentences) and inference (non-MFD sentences) data, which could impact the accuracy of prediction. Nonetheless, we observe that in the absence of explicit moral words, children tend to use more Care/Harm and Fairness/Cheating (individualizing moral foundations) language than Authority/Subversion, Loyalty/Betrayal, and Purity/Degradation language.

**Fig. 3.**
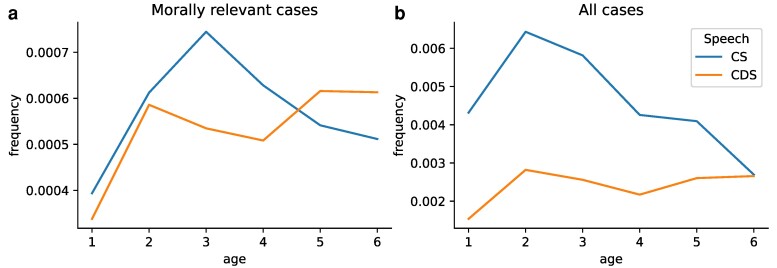
Normalized frequency of property words in child speech and child-directed speech. a) The frequency of morally relevant sentences with property-related vocabulary. b) The frequency of all sentences with property-related vocabulary. The frequencies in both plots are normalized based on the total number of sentences in child speech and child-directed speech. “CS” and “CDS” stand for child speech and child-directed speech, respectively.

Analyzing the moral content in sentences lacking explicit moral language can further be used to uncover interesting communication patterns. For instance, property conflicts happen quite commonly among children and previous studies show that young children can understand property rights (78–83). To investigate how property rights are discussed in child speech and child-directed speech, we use a set of property-related words (*mine, yours, not mine, not yours, n’t mine*, and *n’t yours*) and explore the growth of moral content around these words over time. Figure 3 illustrates the frequency of morally relevant sentences containing property words (Fig. 3a) and the frequency of all sentences containing property words (Fig. 3b). Both plots show the normalized frequencies based on the total number of sentences in child speech and child-directed speech. The results indicate that the overall frequency of property-related words peaks in children at age 2, with morally relevant usages most common at age 3. In both cases, these frequencies decrease over time. Caretakers, however, consistently use these property words with a relatively stable frequency. Examples of property-related moral sentences include: “No, that’s mine” (CS, age 2), “All the money is mine; I tricked you” (CS, age 5), “Here, you can have a sip of mine” (CDS, age 5), or “Yeah, but don’t eat mine” (CDS, age 3). Using our earlier method, we categorize instances of property words into moral foundations. Figure S9 (left) shows that child-directed speech uses property words to convey moral concerns regarding Care/Harm, Fairness/Cheating, and Authority/Subversion almost equally, while child speech focuses more on the Care/Harm moral foundation, followed by Authority/Subversion, Fairness/Cheating, and Loyalty/Betrayal.

Similarly, we explore how obligation modal verbs (*should, must, shouldn’t, mustn’t, should not*, and *must not*) are reflected in the moral language of children and caretakers. Figure S10 shows that the frequency of modal verbs in moral and nonmoral sentences increases gradually in child speech, and compared to children, caretakers use modal verbs much more frequently. We also identify that both caretakers and children tend to express the Care/Harm moral foundation more often than other moral foundations when they use modal verbs in a moral context. Furthermore, children would use obligation modal verbs more commonly for Fairness/Cheating and Loyalty/Betrayal, while caretakers tend to focus on Authority/Subversion, Fairness/Cheating, and Purity/Degradation instead (see Supplementary material for more details).

## Generalization and the emergence of moral foundations in computational models

Our analyses so far have revealed the emergence of the moral foundational language in childhood speech over developmental time. Our findings are based partly on word sense disambiguation, which identifies word usages that we assume to appear in morally relevant contexts. To further validate and understand these moral word usages from childhood speech, here we develop a predictive analysis. Our analysis leverages childhood moral word usages as inputs to train computational models that can automatically infer people’s judgment about moral foundations beyond a child-development setting. Specifically, we explore whether patterns in children’s and caretakers’ moral word usages can be generalized to novel moral scenarios that young children have not encountered.

We apply our model both to predict whether a given utterance is morally relevant (i.e. binary moral relevance prediction) and to predict the principal moral foundation expressed in that utterance (i.e. fine-grained moral foundation prediction). Our objective here is to establish a baseline for assessing the moral relevance and moral foundations of different utterances by solely learning about morality from child and caretaker conversations. We evaluate the generalization of this model against novel datasets that are independent from CHILDES corpora.

To prepare input utterances into compact and computationally manipulable objects, we construct our model using pretrained W2V and GloVe embeddings, which are common models for representing meaning from text (84, 85). To prevent the model from relying on information from rich text or large online corpora, we repeat this process using W2V and GloVe embeddings exclusively trained on the CHILDES dataset. We then build separate logistic regression models for CS and CDS, increasing the number of samples cumulatively with age (from year 1 to year 6). In total, we trained 48 models, accounting for the variation among 6 different ages, 4 word embedding algorithms (pretrained W2V and GloVe and CHILDES-trained W2V and Glove), and 2 types of speech (child and caretaker).

To test the models, we consider three crowd-sourced and well-known moral datasets independent of CHILDES: the Moral Foundations Twitter Corpus (MFTC) (20), the SOCIAL-CHEM 101 dataset (86), and the Moral Foundations Reddit Corpus (MFRC) (87). These datasets represent adult-like language for morally relevant discussions not explicitly covered in child and caretaker conversations. The MFTC is a collection of tweets about various topics, annotated with moral relevance and moral foundation labels. The SOCIAL-CHEM 101 dataset comprises everyday social norms and moral judgments for different Rules of Thumb, annotated with different social labels, including moral foundations. The MFRC consists of Reddit comments annotated with moral foundations and is different from MFTC and SOCIAL-CHEM 101 in three ways. Firstly, each MFRC post is annotated based on six moral foundations, including a partition of Fairness into Equality and Proportionality (88). Since CHILDES conversations are annotated with the original five moral foundations, we replace Equality and Proportionality with Fairness here. Secondly, this dataset does not provide polarity labels for moral foundations, and thus we train our classifiers to predict five moral foundations without distinguishing positive and negative polarities. Thirdly, as indicated in Table S3, each comment in MFRC contains more sentences and tokens compared to MFTC and SOCIAL-CHEM 101. Since we represent each input to the models with the average word embeddings of its tokens, longer comments would be represented less accurately than shorter ones under this setting. To address this issue, we split each comment into its sentences for classification. During inference, our models determine the moral label of each comment in MFRC by taking the majority vote from the predicted labels of its sentences.

**Fig. 4.**
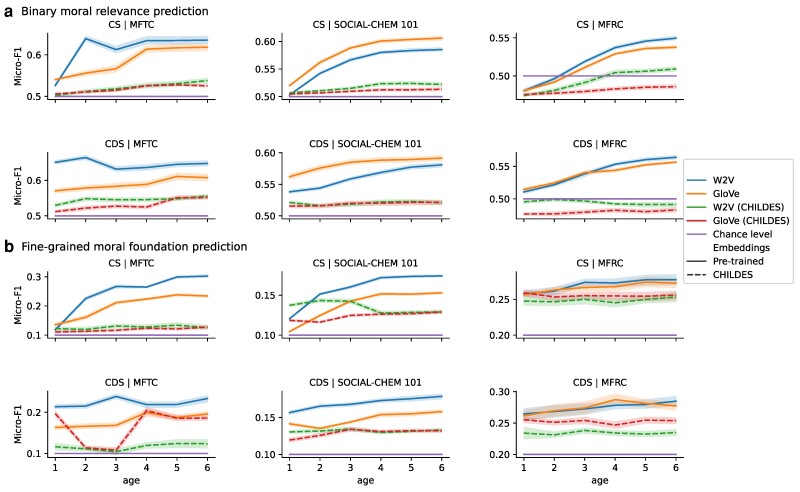
Predictive scores of automated moral inference. The regression models are trained on the embeddings of CHILDES moral sentences to predict moral relevance (a) and moral foundations (b). The results show the performance of these models tested on the datasets of MFTC, SOCIAL-CHEM 101, and MFRC. “CS” and “CDS” stand for child speech and child-directed speech, respectively.

Figure 4 summarizes the outcomes of our analysis on automated inference of moral relevance (Fig. 4a) and fine-grained moral foundations (Fig. 4b) in the MFTC, SOCIAL-CHEM 101, and the MFRC datasets. Since there are two possibilities in the binary moral relevance prediction task and 10 alternative moral foundations (for MFTC and SOCIAL-CHEM 101) in the fine-grained foundation prediction task, the random predictive accuracies are at 0.5 and 0.1 micro-F1, respectively, which is a metric to compute the proportion of correctly classified observations. The random baseline in the fine-grained foundation prediction for the MFRC dataset is 0.2, representing equal distribution among the five moral foundations. Our results show that the models can generalize the moral information learned from CS and CDS to novel scenarios with greater accuracy than a random baseline performing at the chance level. Additionally, we observe that predicting binary moral relevance labels proves more challenging than predicting fine-grained moral foundations in the MFRC dataset. This difficulty may arise from our sentence-splitting strategy, since using a majority vote would struggle to classify posts with only a few moral sentences as morally relevant.

The acquired F1 scores and the significant improvement over the chance level are remarkable in that our models exclusively rely on textual data sourced from children and caretakers without any incorporation of supplementary information, such as books, television shows, facial expressions, gestures, or other nonlinguistic inputs. Moreover, the CHILDES dataset includes a vocabulary much more restricted and simplistic than the vocabulary of the adult moral datasets, which limited the model as well. Despite these limitations, our findings suggest that moral information in CHILDES alone can be generalized for making moral judgment in new scenarios. Consistent with our expectation, the accuracy of these elementary models that are trained on CHILDES data is not as substantial as state-of-the-art language models that are fine-tuned directly on the MFTC, SOCIAL-CHEM 101, and MFRC datasets themselves (20, 86, 87). But the key point demonstrated by our findings is that the patterns in the moral foundational lexicon of childhood speech help to grow moral foundations in a computational model without such information encoded a priori.

In addition to the broad observation above, certain specific comparisons are revealing. In particular, models lacking linguistic knowledge beyond child and caretaker conversations from ages 1 to 6 (i.e. W2V [CHILDES] and GloVe [CHILDES]) have lower predictive performance compared to the models with pretrained word embeddings of W2V and GloVe (see Fig. 4 for comparison between pretrained and CHILDES embeddings). We also observe that logistic regression models trained on MFTC and SOCIAL-CHEM 101 datasets, instead of CHILDES, with pretrained W2V embeddings, have comparable predictive power to our W2V and GloVe models in Fig. 4, with micro-F1 of 0.65 for MFTC and 0.61 for SOCIAL-CHEM 101 in binary prediction, and micro-F1 of 0.61 for MFTC and 0.27 for SOCIAL-CHEM 101 in fine-grained prediction. Table S6 further shows the performance of models trained on MFTC and tested on SOCIAL-CHEM 101 and vice versa. This result shows that generalization over moral language usage with simple logistic regression and word embedding models is a difficult task, even when the training and test datasets come from the same distribution or when both represent adult (vs. child) moral language. This also suggests that using conversations between children and caretakers as a source of training data is as informative as using ground-truth datasets for moral foundation prediction.

Supplementary material provides the confusion matrices for the model prediction using pretrained W2V and W2V (CHILDES) input embeddings. Unsurprisingly, identifying the specific moral foundation (1 out of 10) is more challenging than predicting the moral relevance of a sentence (binary prediction) in most cases. Similar to how moral foundations emerge linguistically in child development, we observe the models to predominantly capture the individualizing moral foundations (Care/Harm, Fairness/Cheating) and Degradation, with the majority of errors occurring when a binding moral foundation is incorrectly predicted to be an individualizing one. Supplementary material also provides examples of successful and unsuccessful prediction samples from the model that used GloVe (CHILDES) input embeddings.

To evaluate whether the utterances we filtered from CHILDES are indeed morally relevant, we compare the predictive power of models trained on the utterances identified as morally relevant to the predictive power of models trained on the remaining utterances in CHILDES. We created different sets of control utterances by random sampling. These sets consist of utterances without a moral seed word, drawn randomly from CHILDES. As shown in Table 2, the models trained on the moral utterances (referred to as Moral train set) generally outperform the same models trained on the random control sets (referred to as Random train set), both for binary moral relevance prediction and fine-grained foundation prediction, specifically when we use pretrained word embeddings. This analysis shows that the set of utterances we identified as morally relevant is indeed more morally informative than the rest of the utterances in CHILDES.

**Table 2.** Predictive scores of automated moral inference in models trained on different sections of CHILDES for binary moral relevance and fine-grained moral foundation prediction.

Test set	Train set	CS			
		W2V	GloVe	W2V (CHILDES)	GloVe (CHILDES)
**Binary moral-relevance inference**					
MFTC	Random	0.499±0.006	0.498±0.005	0.498±0.005	0.499±0.005
	Moral	0.634±0.01	0.618±0.01	0.535±0.007	0.524±0.003
SOCIAL-CHEM 101	Random	0.507±0.006	0.494±0.004	0.495±0.004	0.494±0.003
	Moral	0.585±0.004	0.605±0.004	0.521±0.003	0.513±0.003
MFRC	Random	0.496±0.003	0.494±0.004	0.498±0.003	0.507±0.002
	Moral	0.55±0.003	0.538±0.002	0.509±0.003	0.486±0.003
**Fine-grained moral-foundation inference**					
MFTC	Random	0.1±0.0	0.1±0.0	0.1±0.0	0.1±0.0
	Moral	0.301±0.006	0.238±0.005	0.131±0.01	0.125±0.006
SOCIAL-CHEM 101	Random	0.1±0.0	0.1±0.0	0.1±0.0	0.1±0.0
	Moral	0.174±0.002	0.152±0.002	0.13±0.002	0.13±0.002
MFRC	Random	0.258±0.001	0.257±0.001	0.25±0.001	0.26±0.001
	Moral	0.277±0.008	0.273±0.007	0.253±0.008	0.256±0.007

Test set	Train set	CDS			
		W2V	GloVe	W2V (CHILDES)	GloVe (CHILDES)
**Binary moral-relevance inference**					
MFTC	Random	0.496±0.005	0.485±0.007	0.496±0.005	0.487±0.004
	Moral	0.651±0.009	0.615±0.011	0.542±0.005	0.555±0.007
SOCIAL-CHEM 101	Random	0.497±0.004	0.493±0.005	0.503±0.004	0.493±0.004
	Moral	0.58±0.005	0.59±0.005	0.52±0.004	0.524±0.004
MFRC	Random	0.497±0.003	0.493±0.004	0.497±0.003	0.498±0.003
	Moral	0.564±0.003	0.556±0.002	0.491±0.004	0.483±0.004
**Fine-grained moral-foundation inference**					
MFTC	Random	0.102±0.001	0.1±0.0	0.1±0.0	0.1±0.0
	Moral	0.29±0.007	0.204±0.008	0.114±0.011	0.203±0.011
SOCIAL-CHEM 101	Random	0.1±0.0	0.101±0.0	0.1±0.0	0.1±0.0
	Moral	0.175±0.004	0.152±0.003	0.128±0.003	0.131±0.003
MFRC	Random	0.258±0.001	0.257±0.001	0.25±0.001	0.26±0.001
	Moral	0.285±0.009	0.277±0.008	0.235±0.006	0.254±0.006

The values show 95% confidence interval achieved by using different subsets of randomly selected training samples in each group (all the same size). The model with the best performance is shown in bold font. Random models are trained on randomly selected sentences from CHILDES that do not have any MFD seed words. The Moral models are trained on the morally relevant sentences of CHILDES.

## Discussion and conclusion

Within the extensive literature that describes and discusses the moral foundations, an important open question lingers: when do children start expressing different moral words? To address this question, we present a novel approach that connects moral developmental psychology with scalable computational methods to discover the emerging patterns of the moral foundational lexicon in child language development, especially through the first six years of life in English-speaking children and caretakers.

Our work goes beyond the existing literature on the conceptual development of morality in several ways. First, we review the available evidence to offer an estimated timeline for the conceptual development of moral foundations, which suggests that children typically demonstrate their perceptions of individualizing moral foundations (Care/Harm and Fairness/Cheating) earlier than the binding foundations (Authority/Subversion, Loyalty/Betrayal, and Purity/Degradation).

Second, we conduct a large-scale analysis of child-caretaker conversations and find parallels between the usage of the moral foundational lexicon and the conceptual development of morality in children. Specifically, we find that Care/Harm is the most widely expressed moral foundation in both child and caretaker speech. We further analyze the emergence of all five moral foundations and explore both the positive and negative dimensions of morality in an equal setting. We observe that caretakers tend to express moral concerns for Degradation at a much higher rate than children. Caretakers also place greater emphasis on Fairness, whereas children’s speech represents instances of Cheating more frequently.

Third, we show in a modeling framework that moral utterances in childhood can be used to grow computational models that predict moral relevance and foundations in novel scenarios, consistently in ways that resemble moral judgments by human participants. While our study does not directly weigh in on the debate between nativist and constructivist views toward morality or claims on the intuitiveness of the moral foundations, our findings do indicate incremental acquisition of the moral foundational lexicon through child development.

Our analysis of generalization suggests that the concepts of moral relevance and moral foundations can be learned effectively from moral conversations between children and their caretakers. This finding is particularly revealing because we purposely designed our models to initially contain no moral knowledge beyond child-caretaker conversations, and yet through learning from these linguistic contexts the models can generalize to infer moral relevance and foundations beyond the child speech environments. It suggests that even in the absence of other sources of input, linguistic communication can be helpful for moral judgment toward new and real-world scenarios. To be clear, however, this analysis does not serve to reproduce the accuracy of state-of-the-art models in moral judgment prediction. Rather, our purpose is to demonstrate that it is possible to make moral inferences in new scenarios solely based on linguistic description of morality derived from conversations between young children and their caretakers.

Although our findings shed light on the emerging order of moral foundational lexicon in the first 6 years of life in English-speaking children, they do not necessarily suggest that the same order is representative of all situations and contexts. For instance, certain moral foundations, such as Loyalty/Betrayal, might be more prevalent in conversations between children and their peers, siblings, or teachers (whereas the CHILDES dataset only includes conversations between children and their caretakers). Moreover, children might use nonmoral words, such as *mom* or *dad* to express concerns or anticipation of their parents’ authority earlier than the time our findings suggest. We further acknowledge that our main computational methodology relies on the moral words in MFD, yet there are several cases wherein the moral concern is uttered using nonmoral words (e.g. *mine*, *stop*, and *must*), or manifests gradually throughout the course of a conversation, rather than being articulated within a single sentence. These cases are not fully reflected in the results of our analyses. Our framework also does not identify sentences with multiple moral foundations. For example, parents could enforce rules about sharing toys, which involve both Fairness/Cheating and Authority/Subversion moral foundations. Despite these limitations specific to the present dataset and dictionaries, our methodological approach does offer the opportunity to investigate, in a scalable way, variations in moral language use across contexts and developmental stages.

Research in moral psychology has shown that individuals from different cultural backgrounds (e.g. Western vs. Eastern cultures) or political stances (e.g. liberals vs. conservatives) draw upon different moral foundations (1, 17). For instance, liberals tend to lean on individualistic moral foundations, while conservatives favor binding foundations. In our analyses, we use the English portion of the CHILDES database, collected from North American families and the results may or may not generalize to other languages and cultures. Future work can explore the emergence of moral foundations within families of diverse cultural, linguistic, and political backgrounds. The outcomes of such studies might concur or differ from our findings, which would shed light on how the emergence and gradual changes of moral foundations in child language are influenced by the socio-cultural environment in which a child grows up.

Our experimental survey captures the heterogeneous nature of the Purity/Degradation foundation (56), because moral utterances related to this foundation encompass concepts of both physical and spiritual purity/contamination. For instance, our results suggest that both of the utterances “keep your mouth shut when you have food in it” and “every time you feel like swearing and saying bad words just remember this” are morally relevant and related to the Degradation foundation, but the former expresses concerns about physical contamination while the latter deals with more abstract social forms of contamination. Investigating the emerging order of the heterogeneous conceptualization of purity during child development will be an important direction for future research.

In sum, our work demonstrates that large-scale computational analyses of child speech offer a quantitative and comprehensive documentation of the developmental trends in moral language use. Our approach also builds a new connection between moral language development and computational moral inference. We hope that our study will create new opportunities for better understanding the origins of the moral lexicon.

## Materials and methods

### Child-caretaker speech data

We collected 44 text corpora from the CHILDES database (69), with more than one million transcripts, from which we extracted 380K sentences of child speech (CS) and 626K sentences of child-directed speech (CDS). We tagged each utterance with the age of the child at the time of recording. Other than age, CHILDES includes the child and caretaker gender information. We identified 31K unique utterances in CHILDES that included at least one mention of the MFD seed words from children and caretakers in the age range of 1 to 6. In all our experiments, we removed the utterances from the Hall corpus since they were collected in controlled age, socioeconomic and ethnic groups.

### Data processing and sentence clustering

To preprocess the CHILDES utterances for clustering, we first lower-cased them, split them into sentences, removed punctuation, and lemmatized the remaining tokens. All the preprocessing is done using the NLTK toolkit. We then used SBERT (89)—a state-of-the-art technique from natural language processing—to represent the utterances in a high-dimensional, contextually informed semantic space, and reduced the dimension with principal components analysis to keep 95% of variance. We use distributed semantic vectors so that utterances with similar meanings have proximate representations. We next used a Gaussian mixture model (GMM) to assign the utterances to *k* clusters, whereby a cluster is specified as a Gaussian distribution. The number of clusters *k* ranged from 2 to 10 and is chosen by grid-search to maximize the Silhouette score (90) of the clustering. All implementation was done using the scikit-learn (91) package. We followed this procedure for the positive and negative poles of each moral foundation in CS and CDS utterances separately (positive and negative poles were treated as different moral foundations). As children’s and adults’ speech can be structurally different, overall we trained 20 GMM models, and obtained 103 clusters in total.

### Moral utterance annotation

To determine which clusters are morally relevant vs. not, we conducted a survey to obtain human annotation. The full study protocol was approved by Research Ethics Board of the University of Toronto (ethics protocol #36310), and we obtained informed consent from all participants. In this survey, the participants were asked to (i) determine if a given sentence was spoken in a moral context (for the binary moral relevance label) and (ii) if so, identify the moral foundation(s) expressed in the sentence (for the moral foundation label). We represented each cluster in the survey by deriving 10 prototypical and 10 peripheral sentences (at most), allowing us to annotate a much smaller set of utterances in the survey. The prototypical sentences are the ones with the highest proximity, measured by cosine similarity, to the cluster center (i.e. the average of all the utterances in the cluster) and the peripherals are the furthest to the center, with respect to the cosine similarity of their contextual distributed representations. Equation 1 specifies how the proximity of a sentence *s* to its cluster *C* is measured, where $v_{s}$ is the semantic representation of sentence *s*.


(1) $$\text{Proximity}(s,C)=\cos\;(v_{s},\frac{\sum_{s_{i}\in C}v_{s_{i}}}{\left|C\right|}).$$

In total, we gathered 670 utterances from CDS and 701 utterances from CS. We recruited 300 participants, whose first language was English. Each participant annotated 40 utterances, drawn randomly from the data. We used the Prolific recruitment platform, and the data collection was done through the Qualtrics platform. To ensure inclusion of only highly attentive participants, we used a tricky attention check and removed all participants who failed it (N=50). This resulted in an average number of 7.32 annotators per utterance.

To determine if an utterance in the survey was morally relevant and to prevent the overestimation of the moral language, if at least 75% of the participants annotated an utterance as morally relevant, it was considered moral. The moral foundation of the utterance was then determined by taking the majority vote of the annotations. For example, an utterance like “he’s really not to be trusted very much” was initially regarded as an example of Fairness (because the word *trusted* is a seed word of the Fairness moral foundation). However, the majority vote from the participants was Loyalty, and thus it would be counted as an example of Loyalty. If less than 50% of the participants annotated an utterance as morally relevant, the utterance was considered nonmoral. If between 50% and 75% of the participants annotated the utterance as moral, we counted the utterance as morally relevant only if its initial moral foundation matched that from the majority vote of the annotators; otherwise, the utterance would be counted as nonmoral, given the insufficient agreement among annotators about its moral relevance. In addition, we considered all the following one-word utterances as nonmoral: *mommy*, *mother*, *cried*, *together*, *monarch*, *together*, *food*, *blood*, *mucky*, *trash*, *garbage*, *nurse*, *queen*, *refused*, *country*, *wife*, *daddy*, *dad*, since using them without any other context in an utterance would convey little to no information about the speaker’s moral concerns.

For each cluster, we followed a similar procedure and took the majority vote from the participants’ responses to all the cluster’s utterances in the survey. At the end, for each utterance and each cluster we obtained two labels, one representing the moral relevance and one representing the moral foundations.

We defined the agreement ratio metric as the number of survey utterances in a cluster whose moral annotation agrees with their cluster’s moral label. Among all the clusters, we obtained high average agreement ratios of 0.79 for the moral relevance label and an average agreement ratio of 0.77 for the moral foundations label.

### Further annotation

The survey-based procedure enabled us to derive moral labels for the 1,371 utterances in the survey. We further used these annotations to automatically identify moral labels for the remaining utterances in CHILDES. One potential solution involves annotating the remaining utterances based on moral labels of their respective clusters (92). However, this approach can result in homogeneous labels for all the utterances in a cluster, which may not accurately capture the nuances of the utterances. Instead, we took an alternative approach by annotating each utterance with the majority vote between the moral label of its cluster and the labels of its two nearest neighbor utterances from the survey. These neighbor utterances were the ones from the survey that shared the most semantic similarity with the query utterance.

For both moral relevance and moral foundations, we took the majority vote between the cluster label and the neighbors’ labels. If the cluster and the two neighbors had different moral foundations, resulting in a tie, we annotated the utterance with its initial moral foundation determined by the seed moral word in it. If the initial moral foundation of the utterance was not among the votes, we used the moral foundation of the cluster as the tiebreaker. We tested this process with different numbers of nearest neighbors and found that using only the first two nearest neighbors generates the most reliable results.

To determine whether the frequency of a moral word is insignificant, we used permutation tests. In these tests, we created samples from moral sentences in child speech (with replacements) for each age while ensuring that the size of the samples matched the original data. We then compared the frequency of each moral word with the average word frequency in the sampled sentences. The *P*-value for each word is estimated based on the number of times the word’s frequency in moral sentences is above the average. Using a significance level of α=0.01, we identify words whose frequency is significantly lower than the average word frequency in moral language at a particular age.

For sentences without explicit mentions of MFD words, we annotated them based on their 3-nearest neighbors in the annotated survey. This process differs slightly from annotating sentences with MFD mentions, where we used the 2-nearest neighbors and the cluster information. This is because the non-MFD sentences were not included in the dataset for the clustering model, and thus we cannot assign them to our clusters with high confidence. Instead, for each non-MFD sentence we identified the most semantically similar sentences in the survey (i.e. the 3-nearest neighbors) and then used the majority vote for binary moral relevance and fine-grained moral foundation prediction. In total, we identified 200K unique sentences without MFD words that were classified as morally relevant. To estimate the nearest neighbors, we use sentence representations from the SBERT language model (89).

### Automated moral inference

We identified the morally relevant utterances in CHILDES from the clustering procedures described and trained logistic regression models to predict the moral relevance and the moral foundations in the utterances outside the scope of child-caretaker conversations. The input to the logistic regression models were the semantic representations of the utterances in CHILDES, and the target labels were their moral relevance labels (for binary prediction) or their moral foundation labels (for fine-grained prediction). For the semantic representation of each utterance, we took an average of the semantic representation of the tokens in the utterance, using W2V skip-gram model (84) and GloVe (85) embeddings. In order to prevent our models from relying on the information in adult-like language, we repeated this process by using W2V and GloVe embeddings that we exclusively trained on the CHILDES dataset.

For testing on MFTC and MFRC, we used the majority votes of the annotators for the moral labels of the utterances. We represented the SOCIAL-CHEM 101 utterances as morally relevant if the annotations categorized the utterance in the “morality” group. To test our models, we created ten balanced datasets by randomly sampling utterances from MFTC, SOCIAL-CHEM 101, and MFRC. The number of samples in the train and test sets are shown in Tables S4 and S5.

### Control conditions

We compared the moral information in the utterances identified as morally relevant with the remaining utterances in the CHILDES dataset. To do so, we created different sets of random control utterances. These sets consist of utterances without a moral seed word drawn randomly from the CHILDES dataset. We assigned moral foundations to these utterances randomly and repeated this sampling 20 times for each model and control the number of samples in each category so that they were equivalent to the sample sizes in our initial models. We trained new models on these control sets and compared their performance on the test datasets.

## Supplementary Material

pgae278_Supplementary_Data

## Data Availability

Data and code for replicating our analyses are available at https://osf.io/knu6s/.

## Notes

a The most recent work has split the Fairness foundation into two, namely Equality and Proportionality (2). Liberty/Oppression has also been suggested as an additional foundation (3). Here, we focus on the five foundations that have been most extensively studied in the literature.
