# Supplementary material for: Quantifying the emergence of moral foundational lexicon in child language development
Source: PNAS Nexus. 2024 Aug 20;3(8):pgae278. doi: 10.1093/pnasnexus/pgae278 (PMC11334335; doi:10.1093/pnasnexus/pgae278)
Supplement: pgae278_Supplementary_Data [file pgae278_supplementary_data.pdf]

Supplementary Material for

Quantifying the emergence of moral foundational lexicon in child language development

Aida Ramezani, Emmy Liu, Spike W. S. Lee, Yang Xu

Aida Ramezani.
E-mail: armzn@cs.toronto.edu

This PDF file includes:

Supplementary material text
Figs. S1 to S12
Tables S1 to S7
References

Contents

A Moral foundational lexicon . . . 3
B Negation in moral utterances . . . 3
C Frequency and the Age of Acquisition of the moral foundational lexicon . . . 3
D Mean Length of Utterance . . . 3
E Property words and modal verbs . . . 3
F Error analysis . . . 3
G Sample sizes . . . 4
H Cross validation . . . 4
I Test cases . . . 4

List of Figures

S1 Word cloud plots for the moral foundational lexicon . . . 5
S2 Test of significance . . . 6
S3 Negation in child speech . . . 7
S4 Negation in child-directed speech . . . 8
S5 Age of Acquisition . . . 9
S6 Mean Length of Utterance . . . 10
S7 Emergence of implicit moral language . . . 11
S8 Word cloud plots for the non-MFD lexicon . . . 12
S9 Property words and modal verbs in moral foundations . . . 13
S10 Obligation modal verbs . . . 14
S11 Moral foundational lexicon in older children . . . 15
S12 Error analysis plots . . . 16

List of Tables

S1 CHILDES sub-corpora . . . 17
S2 Sample sizes from older children . . . 18
S3 Token statistics . . . 19
S4 Sample sizes in fine-grained moral foundation prediction . . . 20
S5 Sample sizes in binary moral relevance prediction . . . 21
S6 Cross validation . . . 22
S7 Prediction examples . . . 23

Supplementary Material Text

A. Moral foundational lexicon. Table S1 shows the total number of sentences and tokens in each sub-corpora within the CHILDES dataset. Figure S1 provides word cloud plots for the moral foundational lexicon in the CHILDES dataset, including both child and caretaker speech, with respect to their frequency. The plots are generated using the `wordcloud` package. The sizes of the words are correlated with their frequencies in each moral foundation. Words such as *help*, *hurt*, *fair*, *lying*, *police*, *impolite*, *player*, *together*, *enemy*, *food*, *clean*, and *dirty* are the most frequent words from all moral foundations.

Figure S2 shows the percentage of words above the significant level of $\alpha = 0.01$ in each moral foundation for child speech. Words other than the ones shown in this figure are those whose frequencies are statistically insignificant when we compare them to the average frequencies of all the other words present in the moral language sampled from child speech a particular age. As illustrated in the figure, Care/Harm foundation has the highest percentage of words with significant frequency signals. We also observe that while some words in the Authority/Subversion, Fairness/Cheating, and Loyalty/Betrayal moral foundations appear in child speech at ages 1 and 2, almost all of them appear far less frequently than the words in Care/Harm moral foundations. For Purity/Subversion, we find the word *dirty* to appear with statistical significance even at age 1, however, upon closer manual examination, we observe that this word mainly appears in non-moral contexts about physical cleanliness/dirtiness that our algorithm classified as moral.

B. Negation in moral utterances. Figure S3 and S4 show the normalized frequency of positive and negative utterances in each moral foundation for CS and CDS respectively. The negative utterances include at least one of the negative words *no*, *not*, and *n't*, while the positive utterances include none. Children tend to use the negated form of the utterances in Fairness and Authority more than other foundations. For example, the negative form of the word *fair*, as in "it's not fair" appears more frequently than its positive form in CS. Caretakers in comparison, use much less negation.

C. Frequency and the Age of Acquisition of the moral foundational lexicon. Figure S5 shows that the overall frequency and the Age of Acquisition (AoA) of moral words are different from the emerging order of the moral foundational lexicon in child moral speech. To create these plots, we considered the occurrences of moral words in both morally relevant and morally irrelevant utterances. For example, the utterances "let's go to the fair" and "it's not fair" are both considered occurrences of the word *fair*. We then plotted the average frequency of words in each moral foundation on the left, and the Age of Acquisition (AoA) of these words on the right, both from CS. The bars show the average value over all the moral words in each foundation, and black lines represent the 95% confidence intervals.

For example, at age 2, Figure S5 (left) shows that words in the Loyalty foundation (which is a binding foundation) are used more frequently than the words in the Fairness/Cheating foundation (which is an individualizing foundation). However, we showed in the main text that Fairness/Cheating moral foundations emerge earlier than Loyalty in child moral speech, suggesting that the raw frequency of the word usage in CHILDES is not a counterargument to our results. Figure S5 (right) also shows the Age of Acquisition (AoA) of moral words of different moral foundations, collected from the AoA ratings by Kuperman et al., 2012 (1). The results indicate that the AoA ratings of moral words have a different order from the emerging order of the moral foundational lexicon. For example, the AoA for Authority/Subversion words is higher than for Loyalty/Betrayal words, even though Authority/Subversion emerged earlier both in linguistic development and conceptual development of moral foundations. These findings distinguish the linguistic emergence of the moral foundations from the frequency and the Age of Acquisition of the moral foundational lexicon.

D. Mean Length of Utterance. Figure S6 shows the Mean Length of Utterance (MLU) for the moral sentences in CHILDES. The left plot visualizes how sentence length grows over time in child speech and child-directed speech. The right plot displays the length of the moral sentences for each moral foundation in child speech. We observe that moral words in Care/Harm and Purity/Degradation foundations are typically uttered in shorter sentences and Authority/Subversion contains longer sentences. The sentences in the Fairness/Cheating moral foundation are relatively longer than other foundations in younger children, but their length remains relatively stable over time.

E. Property words and modal verbs. We use the words *mine, yours, not mine, not yours, n't mine*, and *n't yours* to explore the moral language around the property-related conversations and the modal verbs *should, must, shouldn't, mustn't, should not*, and *must not* to study how obligation is expressed in moral language. Figure S10 shows the frequency of obligation model verbs in moral sentences (left plot) and all sentences (right plot) in CHILDES. Both plots are normalized according to the total number of sentences in child and child-directed speech. Figure S9 further categorizes property words (left) and modal verbs (right) into different moral foundations. The frequencies in each plot are normalized to sum up to 1 in CS and CDS separately.

F. Error analysis. Figure S12 provides the confusion matrices for the pre-trained W2V and W2V (CHILDES) models in the fine-grained moral foundation prediction task at age 6. The rows represent the target labels, and the columns represent the predicted labels. Similar to how moral foundations emerge linguistically in child development, our models also predominantly reflect the individualizing moral foundations (Care/Harm, Fairness/Cheating) and Degradation, with the majority of errors occurring when a binding moral foundation is incorrectly predicted to be an individualizing one. The values are normalized to have a sum of 1 in each row. The higher values are shown with brighter colors.

G. Sample sizes. Table S2 shows the total number of utterances in CHILDES from child speech and child-directed speech regarding children older than six years old. The number of utterances for older children decreases drastically with age, and the data is much sparser than the available utterances in CHILDES from younger children, as shown in Table S5.

Table S3 provides the average number of tokens per post and sentence for datasets of adult moral language. The number of samples in the train and test sets for our automated inference analysis is shown in Table S4 for fine-grained moral foundation prediction and Table S5 for binary moral relevance prediction.

H. Cross validation. Table S6 shows the performance of models trained and tested on MFTC and SOCIAL-CHEM 101. Each entry shows the average aggregated results from 10 different samplings of the testing datasets, which are shared among the models in this table and the models trained on the CHILDES dataset so that all models are evaluated under the same setting.

I. Test cases. Table S7 provides examples of successful and unsuccessful test samples for the model that used GloVe (CHILDES) input embeddings at age 6. For instance, this model accurately predicts that the moral foundation of the utterance "It's good to take proper care of your pet" (from the SOCIAL-CHEM 101 dataset) is Care. However, it fails to identify the moral foundation of the sentence "It is wrong to be disrespectful to your mother", which is incorrectly predicted to be Care instead of Authority.

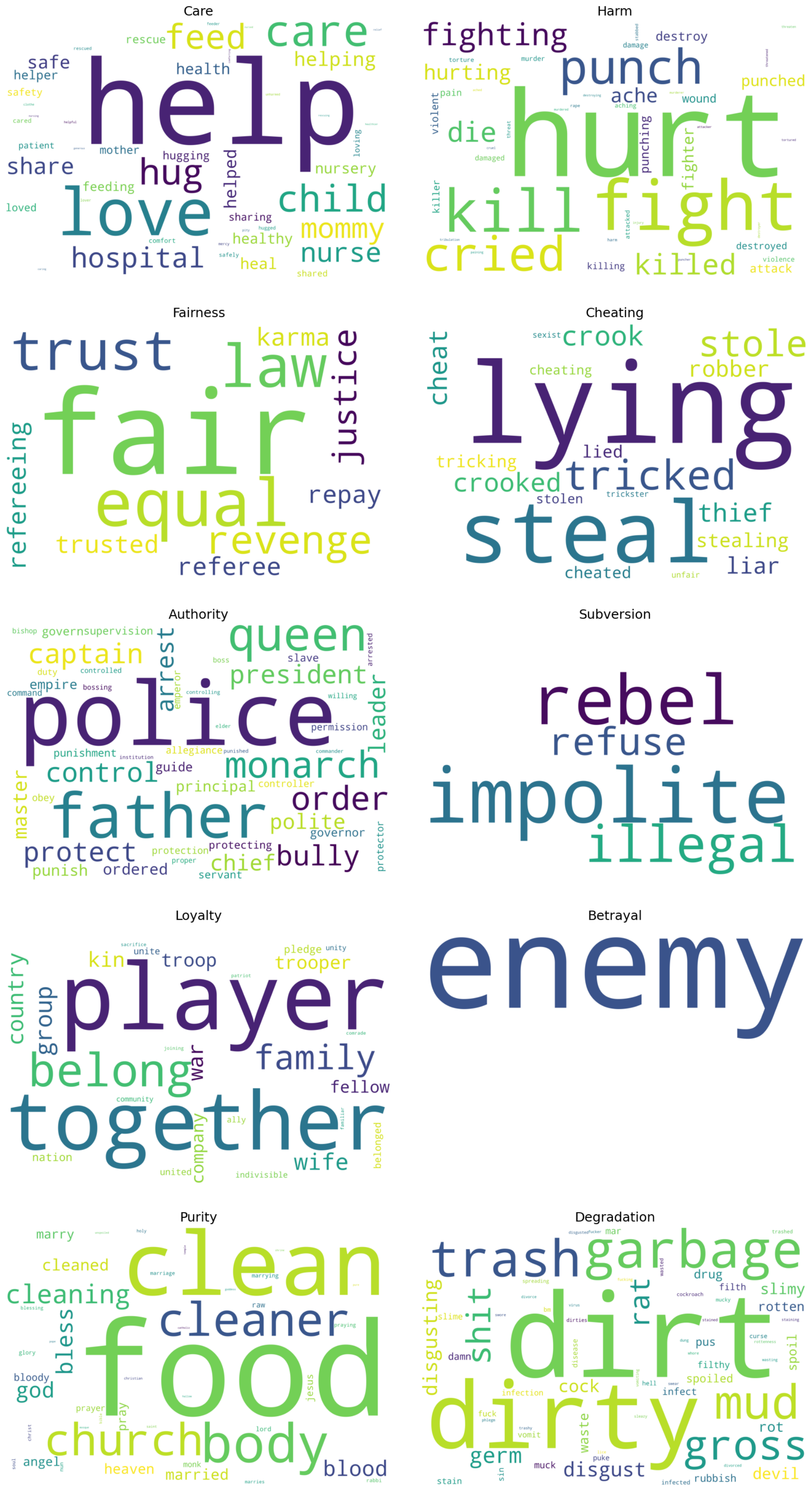

Fig. S1. Word cloud for the moral foundational lexicon in the CHILDES dataset. The size of the words are correlated with the frequency in the moral utterances.

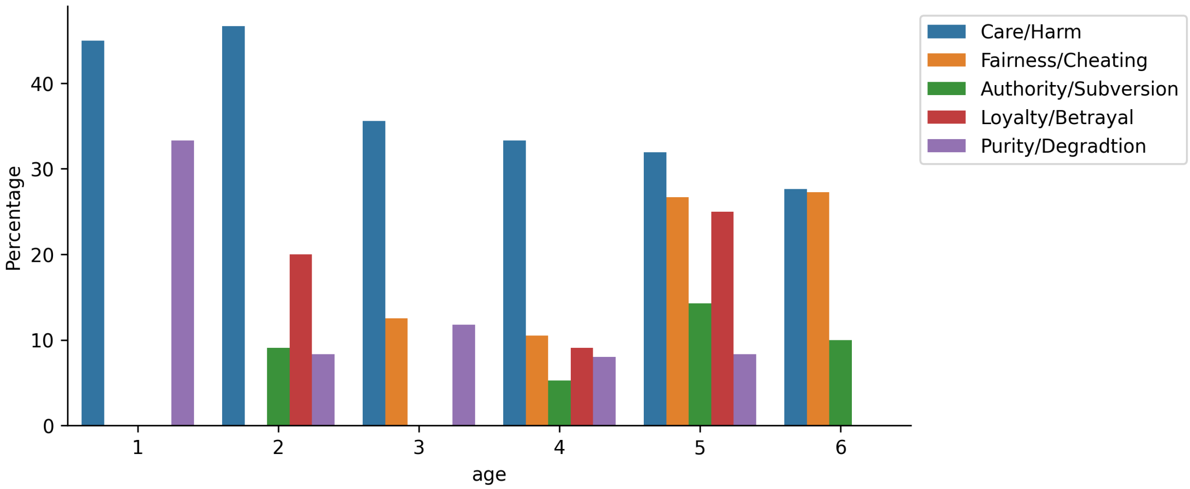

Fig. S2. The percentage of words with frequencies above the average frequency of words in each moral foundation in child speech ($\alpha = 0.01$).

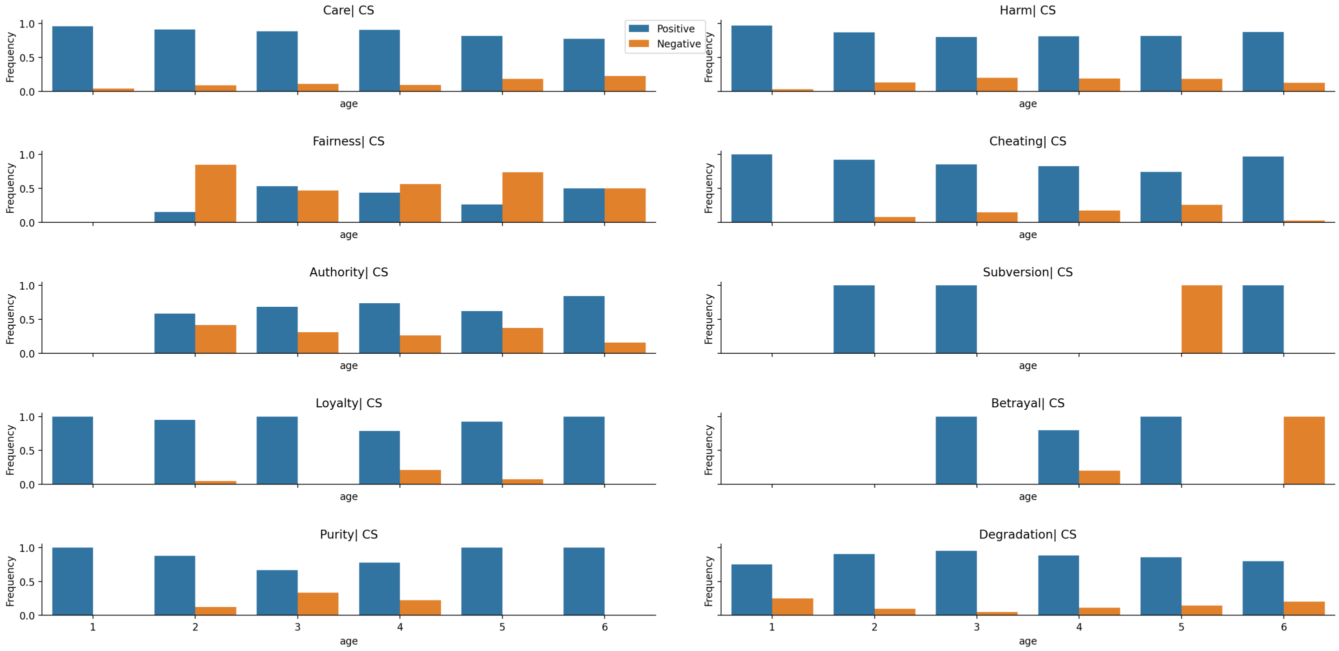

Fig. S3. The normalized frequency of the positive and negative morally-relevant utterances in each moral foundation as spoken by children. The Negative utterances are the ones that mention *no*, *not* or *n't*. CS refers to child speech.

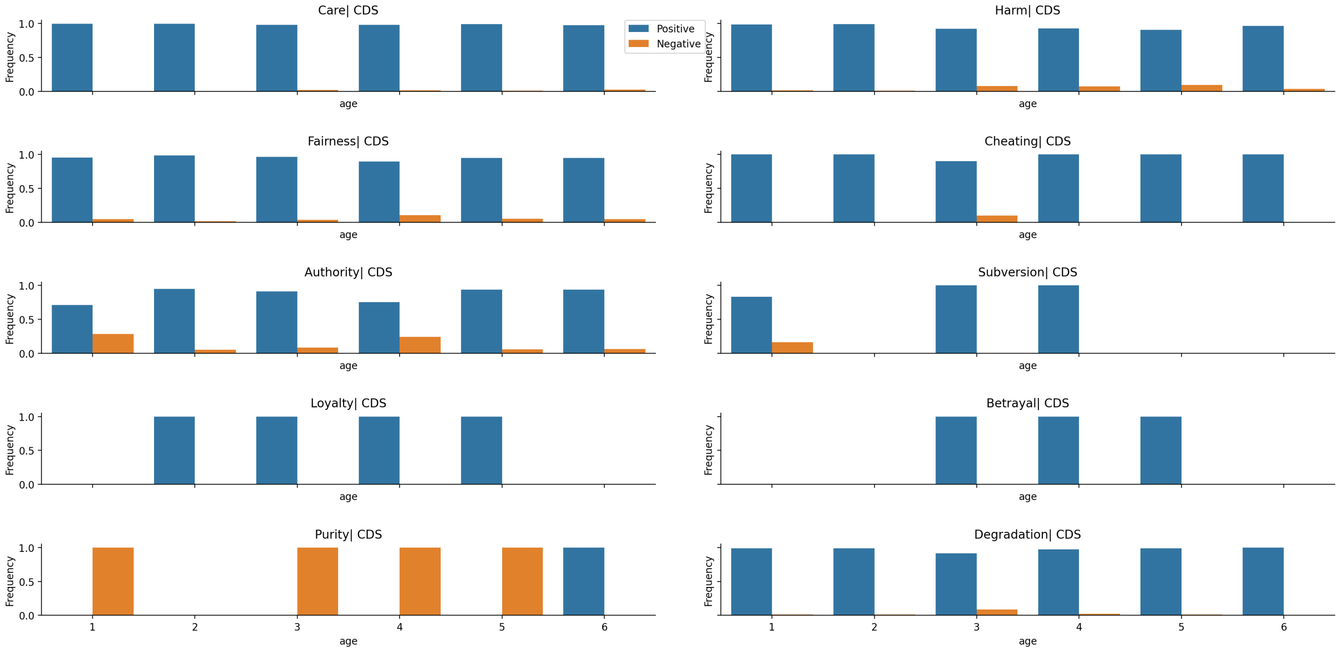

Fig. S4. The normalized frequency of the positive and negative morally-relevant utterances in each moral foundation as spoken by caretakers. The Negative utterances are the ones that mention *no*, *not* or *n't*. CDS refers to child-directed speech.

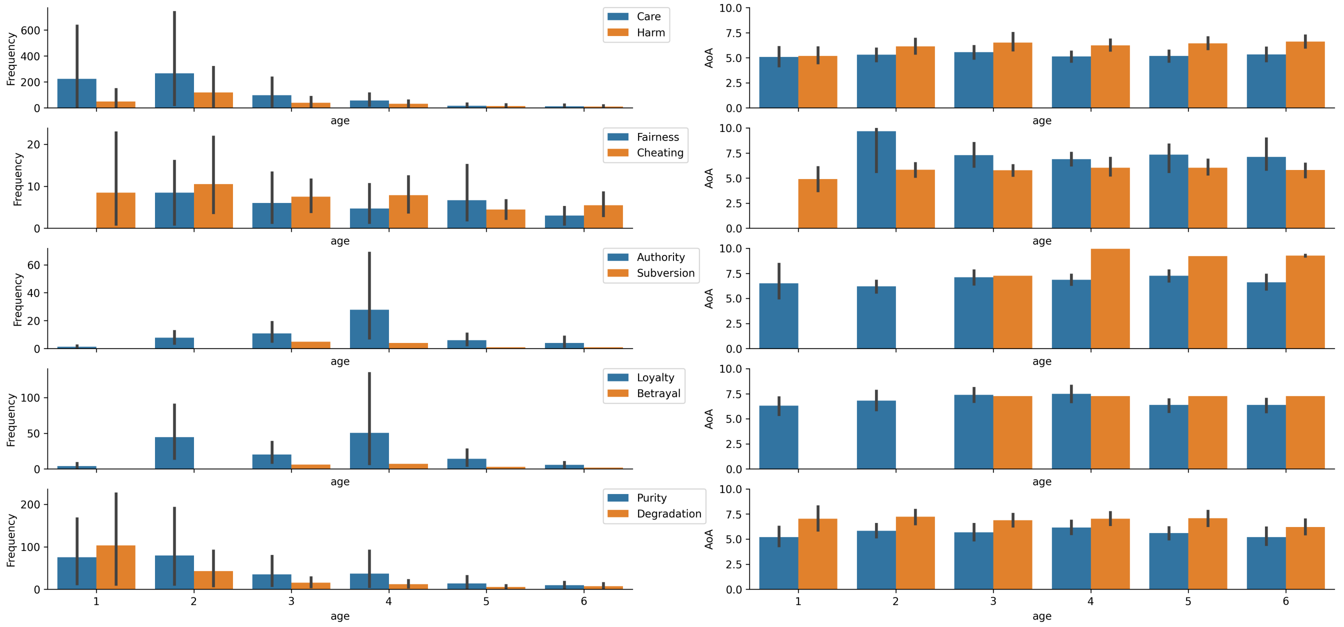

Fig. S5. The frequency and the Age of Acquisition for the moral foundational lexicon in CHILDES child speech (CS). The bars show the average value and the 95% confidence intervals.

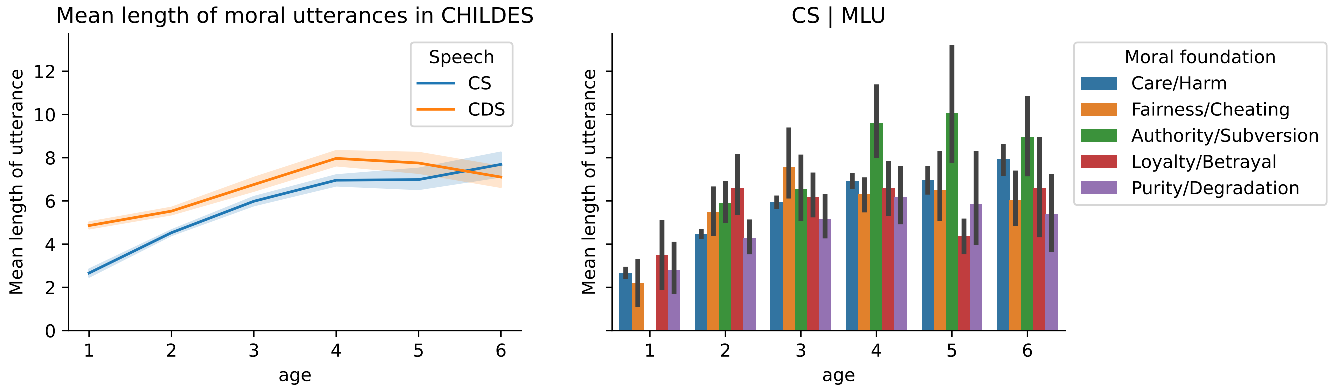

Fig. S6. Differences in the Mean Length of Utterance (MLU) in moral utterances. Left: The MLU for moral utterances in child speech (CS) and child-directed speech (CDS) over time. Right: The MLU of individual moral foundations in child speech. The error markers in both plots show the 95% confidence intervals.

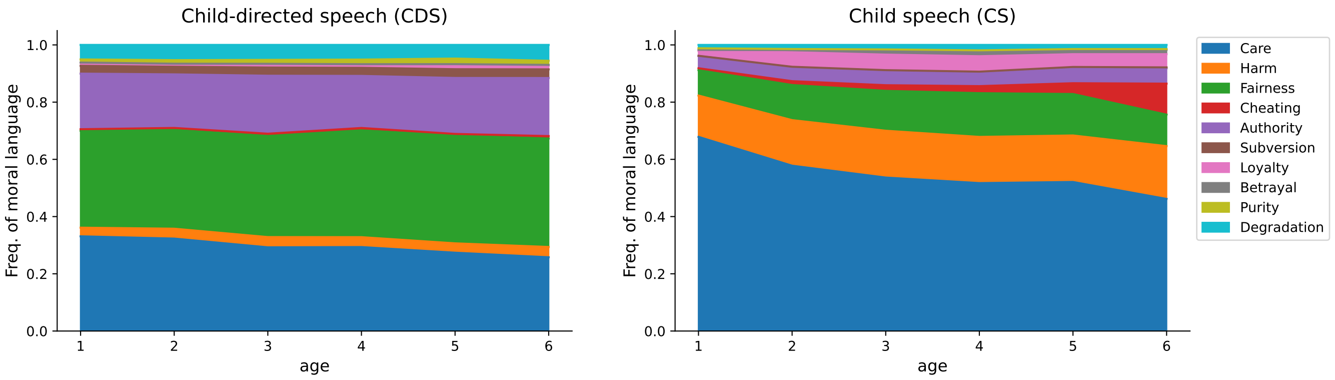

Fig. S7. Stack area charts showing the normalized frequencies of moral foundational language without explicit mentions of moral words in child-directed speech (CDS) and child speech (CS) over time.

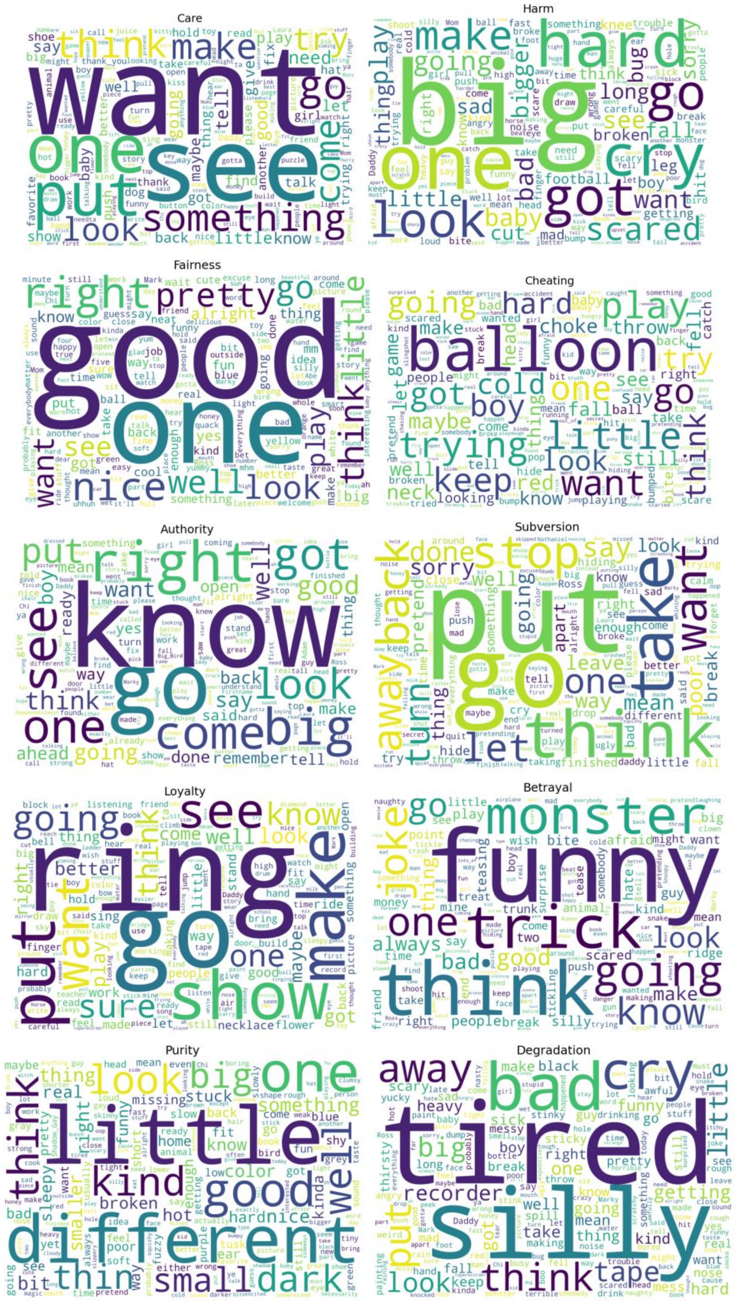

Fig. S8. Word cloud for the non-MFD lexicon in morally relevant sentences of the CHILDES dataset. The sizes of the words are correlated with the frequency in the moral utterances.

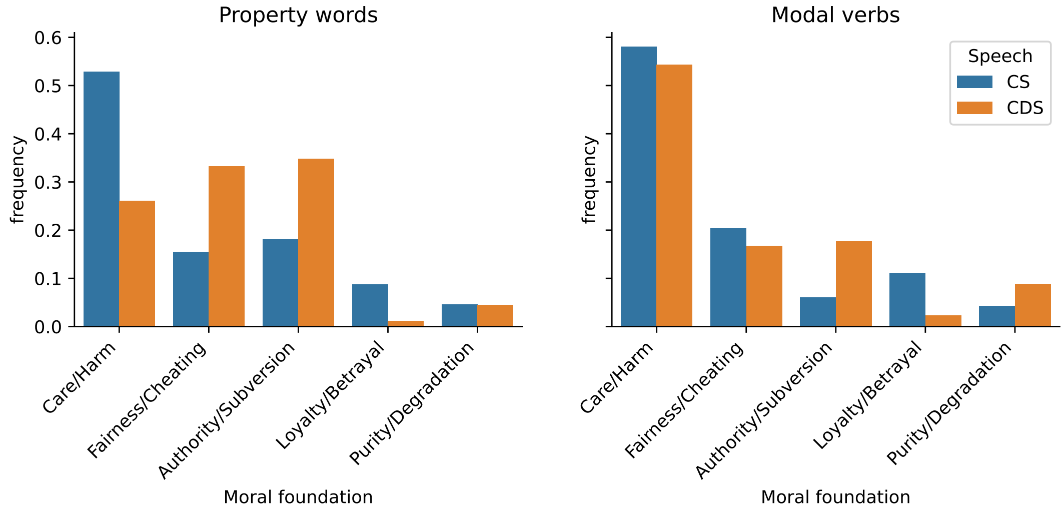

Fig. S9. Frequency of property words (left) and modal verbs (right) in different moral foundations. The frequencies in each plot are normalized to sum to 1 in child speech (CS) and child-directed speech (CDS) separately.

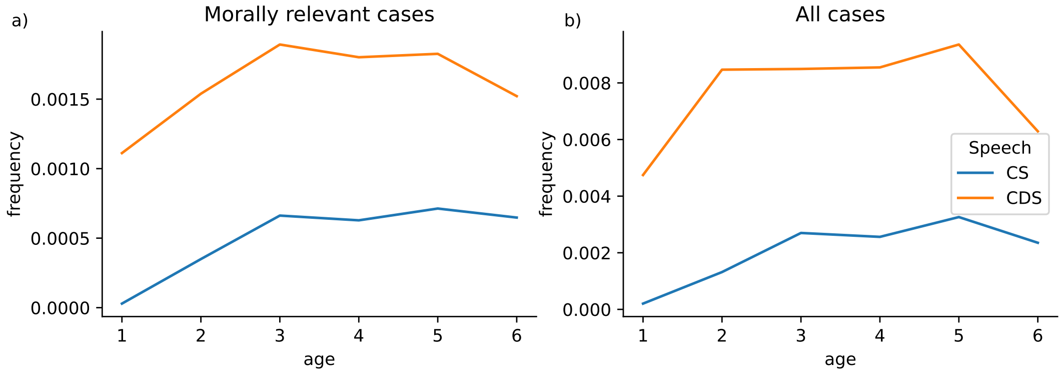

Fig. S10. Normalized frequency of obligation modal verbs in child speech and child-directed speech. The left plot shows the frequency of morally-relevant sentences with modal verbs. The right plot shows the frequency of all sentences with modal verbs. The frequencies in both plots are normalized based on the total number of sentences in child speech (for the blue lines) and child-directed speech (for the orange lines).

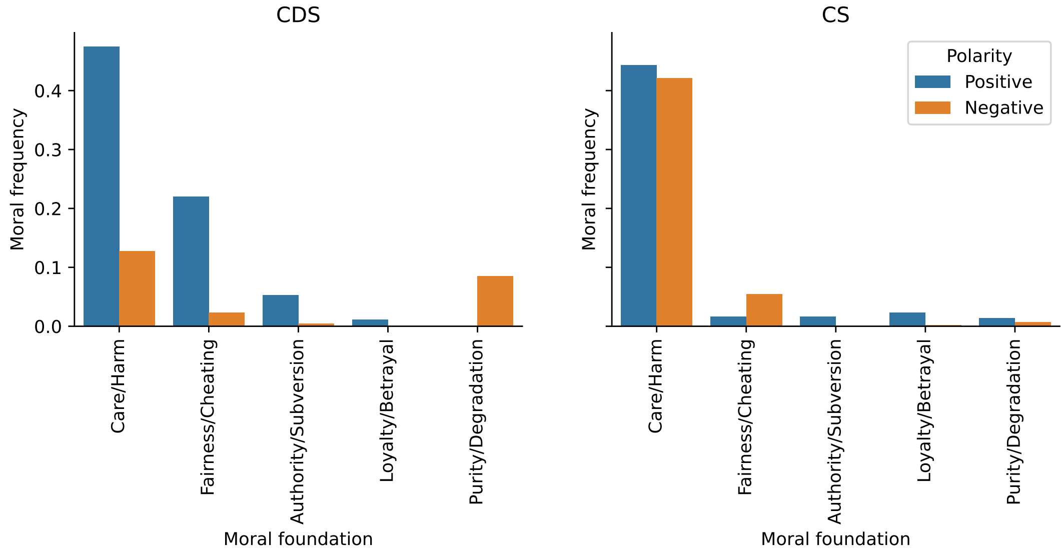

Fig. S11. The frequency of the moral foundational lexicon in child-caretaker conversations from children aged 7 to 11. CDS and CS represent child-directed speech and child speech respectively.

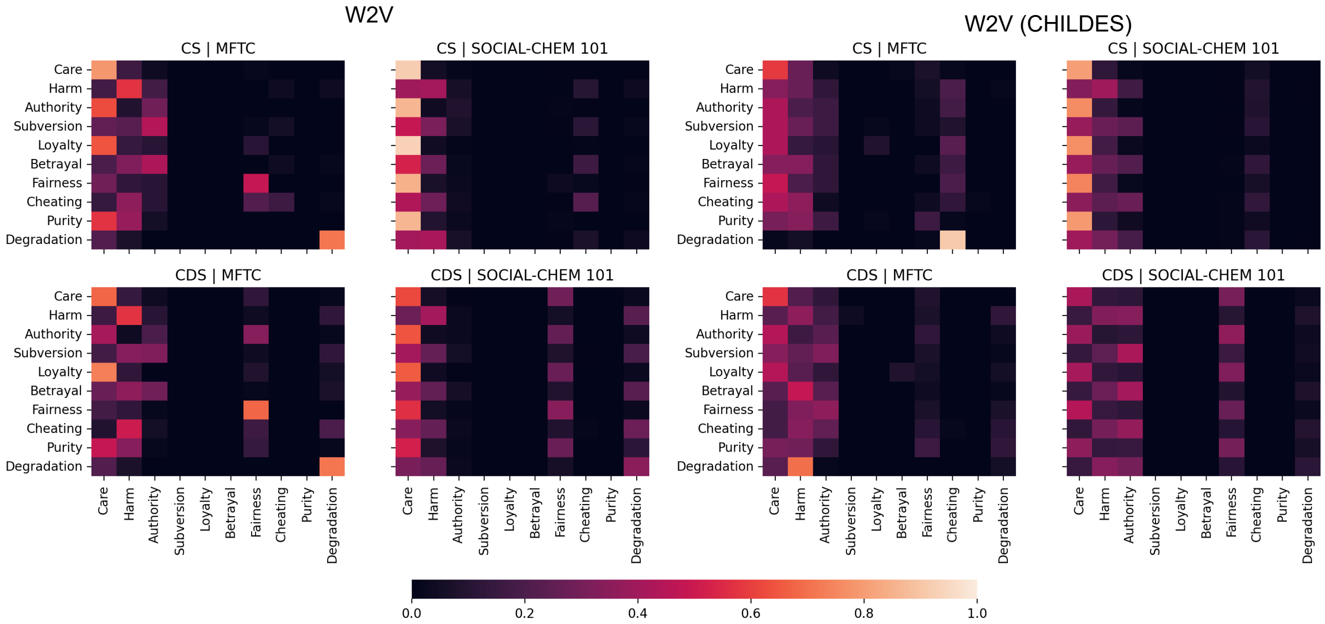

Fig. S12. Normalized confusion matrix from the models with W2V-transformed (left) and W2V (CHILDES)-transformed (right) input at age 6. The columns show the predicted moral foundations and the rows display the target moral foundations. The higher values are shown with brighter colors.

Table S1. Number of sentences and tokens in each sub-corpora in the CHILDES database.

Corpus	Sentence size	Token size
Bates	8,059	34,426
Bernstein	8,778	47,589
Bliss	1,263	5,214
Bloom	22,405	92,007
Bohannon	2,827	11,570
Braunwald	48,529	201,401
Brent	71,719	340,078
Brown	123,033	530,181
Clark	15,252	80,395
Demetras1	11,104	54,487
Demetras2	15,181	68,702
Evans	475	2,192
Feldman	6,791	31,000
Garvey	4,543	25,198
Gelman	107,672	570,261
Gleason	42,336	219,360
Gopnik	2,702	14,079
HSLLD	1,48,430	854,016
Haggerty	1,490	8,322
Hall	85,527	481,431
Hicks	5,229	33,509
Higginson	9,493	42,662
Kuczaj	49,833	277,047
MacWhinney	72,744	421,970
McCune	14,828	52,934
McMillan	147	631
Morisset	18,339	76,798
Nelson	3,700	16,039
NewEngland	30,843	130,585
NewmanRatner	100,676	593,007
Peters	24,862	105,467
PetersonMcCabe	4,609	34,242
Post	20,525	100,030
Rollins	10,294	47,471
Sachs	20,071	83,351
Snow	25,238	119,045
Soderstrom	18,018	85,437
Suppes	51,251	259,963
Tardif	12,844	52,663
Valian	26,673	127,528
VanHouten	6,213	26,110
VanKleeck	4,992	21,709
Warren	7,460	36,297
Weist	67,161	399,014

Table S2. The number of samples in CHILDES in child speech (CS) and child-directed speech (CDS) collected from conversations with children above the age of 6.

	Age 7	Age 8	Age 9	Age 10	Age 11
CS	654	217	294	136	54
CDS	860	39	288	143	11

Table S3. Number of tokens and sentences in the adult moral language datasets. The first row shows the average number of tokens per post. The second row shows the average number of sentences per post, and the third row shows the average number of tokens per sentence.

	MFTC	SOCIAL-CHEM 101	MFRC
Average number of tokens per post	20.55	12.09	41.23
Average number of sentences per post	1.70	1.00	2.68
Average number of tokens per sentence	12.32	12.09	15.69

Table S4. Number of samples in train and test sets for fine-grained moral foundation prediction.

Train											
Dataset	Age	Care	Harm	Fairness	Cheating	Authority	Subversion	Loyalty	Betrayal	Purity	Degradation
CHILDES CS	1	3407	379	0	5	0	0	2	0	3	4
	2	8393	2139	14	50	26	3	20	0	47	69
	3	2973	821	15	68	35	2	17	3	3	48
	4	1242	692	14	94	71	0	14	5	11	28
	5	363	275	23	41	38	1	13	1	2	14
	6	284	215	6	44	19	1	5	2	5	5
CHILDES CDS	1	3606	347	57	4	7	6	0	0	0	357
	2	2958	956	72	9	19	0	2	0	0	102
	3	693	790	67	10	28	3	1	1	1	73
	4	625	1272	58	8	48	1	2	1	3	373
	5	884	407	19	3	17	0	1	2	1	703
	6	131	116	21	6	16	0	0	0	1	34
Test											
MFTC		56	56	56	56	56	56	56	56	56	56
SOCIAL-CHEM 101		500	500	500	500	500	500	500	500	500	500
MFRC		169		169		169		169		169	

Table S5. Number of samples in train and test sets for binary moral relevance prediction.

Dataset	Age	Morally-relevant	Morally-irrelevant
Train			
CHILDES CS	1	3,800	3,800
	2	10761	10761
	3	3,985	3,985
	4	2,171	2,171
	5	771	771
	6	586	586
CHILDES CDS	1	4,384	4,384
	2	4,118	4,118
	3	1,667	1,667
	4	2,391	2,391
	5	2,037	2,037
	6	325	325
Test			
MFTC		815	815
SOCIAL-CHEM 101		3000	3000
MFRC		2182	2182

Table S6. The performance of models trained and tested on moral datasets of MFTC and SOCIAL-CHEM 101.

Binary moral relevance prediction

Train dataset	Test dataset	Micro-F1
MFTC	MFTC	0.649
MFTC	SOCIAL-CHEM 101	0.529
SOCIAL-CHEM 101	SOCIAL-CHEM 101	0.647
SOCIAL-CHEM 101	MFTC	0.590

Fine-grained moral foundation prediction

Train dataset	Test dataset	Micro-F1
MFTC	MFTC	0.612
MFTC	SOCIAL-CHEM 101	0.217
SOCIAL-CHEM 101	SOCIAL-CHEM 101	0.273
SOCIAL-CHEM 101	MFTC	0.297

Table S7. Successful and failed test cases of GloVe (CHILDES) model trained on child speech input at age 6.

MFTC		
Test sample	Correct MF	Predicted MF
Public defender wanted to help #FreddieGray in life, now in death by closing pending criminal charge	Care	Harm
Peace and love to ALL! #ONERACE #AllLivesMatter	Care	Care
INNOCENT PEOPLE DO NOT DESERVE TO DIE BECAUSE OF STEREOTYPES OF RACE OR OCCUPATIONS!! #AllLivesMatter	Harm	Care
Shannon J. Miles is a hate-filled racist murdering coward who committed a hate crime. #BlackLivesMatter	Harm	Harm
If your gonna fight racism and injustice don't fight it with racism and injustice #AllLivesMatter	Fairness	Harm
End injustice	Fairness	Fairness
#blacklivesmatter Never disrespect it... #SandraBland No justice, no peace	Cheating	Harm
Where justice is denied... #AllLivesMatter #MillionsMarchNYC #BlackLivesMatter #EricGarner	Cheating	Cheating
@chucktodd as a conservative it is important to point out great leadership despite party affl @CoryBooker is a true leader with integrity.	Authority	Care
Police Chief: Respecting Authority Means Respecting Protesters	Authority	Authority
CPD's disrespect of Black ppl's humanity is the problem, not disrespect of CPD's authority. #BlackLivesMatter #FTP	Subversion	Fairness
Standing in solidarity against injustice. #blacklivesmatter #alllivesmatter #millionsmarchLA	Loyalty	Cheating
@crowleycnn If u love our military. U can't vote 4 COWARD OBAMA. Media is covering up for this coward.He cud of saved 4 Americans #Sandy FEMA	Betrayal	Care
Believers! Be upright for #Allah, bearers of witness with #justice, let not hatred of a people incite you to not act equitably #Quran 5:8	Sanctity	Care
Black lives are of sacred worth. #blacklivesmatter @unitedchurch @justice_ucc	Sanctity	Sanctity
homosexuality is a sin #blacklivesmatter #africanamerican #iuic #bible #hispanictwitter #Blackjesus #blacktwitter	Degradation	Care

SOCIAL-CHEM 101		
It is good when parents encourage their kids and teach them that they can succeed in school.	Care	Harm
It's good to take proper care of your pet.	Care	Care
It's despicable to try to blackmail any of your spouse's friends.	Harm	Care
It's not okay to disrespect someone after their passing.	Harm	Harm
It's nice to let your classmates borrow writing utensils.	Fairness	Care
It's wrong to discriminate based on race.	Cheating	Harm
You shouldn't bribe family members.	Cheating	Cheating
If someone is doing something they should be kicked off a bus for you should let the driver know even if it is your friend.	Authority	Harm
It is wrong to be disrespectful to your mother.	Subversion	Care
You should always be there for your friends.	Loyalty	Care
Family should stick together.	Loyalty	Loyalty
It's wrong to not be supportive of family members.	Betrayal	Care
It is good to be healthy.	Sanctity	Care
It's wrong to have an open relationship.	Degradation	Fairness
Littering is wrong.	Degradation	Degradation

References

1. V Kuperman, H Stadthagen-Gonzalez, M Brysbaert, Age-of-acquisition ratings for 30,000 english words. *Behav. Res. Methods* **44**, 978–990 (2012).
